# Disinfection with chlorhexidine is more effective than ethanol for buttonhole cannulation in arteriovenous fistula: a randomized cross-over trial

**DOI:** 10.1186/s12882-025-04230-z

**Published:** 2025-07-19

**Authors:** Karin Staaf, Vendela Scheer, Lena Serrander, Anders Fernström, Fredrik Uhlin

**Affiliations:** 1 Department of Health, Medicine and Caring Sciences, Linköping University, Linköping, Sweden; 2 Department of Nephrology, Linköping University Hospital, Region Östergötland, Linköping, Sweden; 3 Department for Biomedical and Clinical Sciences, Linköping University, Linköping, Sweden; 4 Department of Clinical Microbiology, Linköping University Hospital, Region Östergötland, Linköping, Sweden; 5 Department of Health Technologies, Tallinn University of Technology, Tallinn, Estonia

**Keywords:** AV fistula, Buttonhole, Cannulation, Disinfection, Haemodialysis

## Abstract

**Background:**

A patient’s normal skin flora is most often the origin of arteriovenous fistula (AVF) infections. When the buttonhole cannulation technique is used, the risk of these types of infections increases. The most effective disinfectant should be used to prevent AVF infections, but evidence on which to choose is lacking. The present study assessed whether chlorhexidine is more effective than ethanol in patients treated with haemodialysis via AVF.

**Methods:**

In this randomized, cross-over, clinical trial, we compared 5 mg/mL chlorhexidine in 70% ethanol to 70% ethanol alone with and without arm washing using serial sampling of normal skin flora directly before disinfection, immediately after the disinfectant effect time, and 2 and 4 h after disinfection during four different dialysis treatments. Scabs from the buttonhole tracks were collected and the type of bacteria in the scabs compared to the patient’s normal skin flora. All participants were sampled during all four interventions. The CFU/mL and types of bacteria were compared between intervention groups.

**Results:**

Compared to ethanol, chlorhexidine resulted in fewer positive haematin plates directly after disinfection (1.4% vs. 10.8% *p* = 0.032). After 2 and 4 h, ethanol in combination with arm washing showed an increased regrowth of bacteria compared to chlorhexidine without arm washing (60 vs. 170 CFU/mL, *p* = 0.046 and 160 vs. 338 CFU/mL, *p* = 0.022). Scabs from the buttonhole track contained normal skin flora (Cohen’s kappa = 0.97).

**Conclusion:**

Disinfection with chlorhexidine is more effective than ethanol when the buttonhole cannulation technique is used for AVF.

**Trial registration:**

The study was registered June 12, 2023 in Clinical Trials Information System (CTIS) identification number: 2023-505935-11-00.

**Supplementary Information:**

The online version contains supplementary material available at 10.1186/s12882-025-04230-z.

## Background

Well-functioning access to the bloodstream is essential in haemodialysis. There are three possible types of access: arteriovenous fistula (AVF), arteriovenous graft (AVG), and central venous catheter (CVC). AVF is the most common and preferred access due to fewer complications and longer patency [1].

Guidelines describe three possible techniques for cannulating an AVF: rope ladder (RL), area puncture (AP), and buttonhole (BH). When RL is used, a new puncture site is created each time and the whole length of the AVF is used. When AP is used, cannulators create new puncture sites each time, but they are placed in the same area, which is rarely larger than 2–3 cm in diameter. When BH is used, the cannulation is made in exactly the same track at the same angle each time. To form a tunnel track, sharp needles are used. After the track is created, cannulation can be done with blunt needles [2]. The different cannulation techniques are used to varying extents worldwide. For example, in Europe, AP and RL are most frequently used (66% vs. 28%) [3], whereas in Sweden BH is the most popular technique (84%) [4].

European, Australian, and American guidelines recommend RL as the first-choice cannulation technique because BH has shown an increased risk of infection [1, 5, 6]. The risk of infection is recurrent in research including BH [7–10], but there are also studies showing a low infection rate [11–14]. Furthermore, a recent Swedish registry study comparing cannulation techniques and complications did not find any differences in the number of AVF-related infections [4]. Fielding et al. highlights cannulation in AVF as a complex intervention with multiple components that may affect outcome [15]. Differences in the performance of the cannulation process may therefore affect outcome [16].

The AVF infection studies that compare cannulation techniques have differences regarding the described hygiene routines and type of disinfectant used, as well as differences in the infection frequencies. Therefore, many questions on this matter remain to be answered. Factors that increase the risk of AVF-related infections have scarcely been studied.

The patient’s normal skin flora is most often the origin of AVF infections. Colonization by *Staphylococcus aureus* in patients on haemodialysis via CVC or AVF is quite common, and swabs from the nasal mucosa have demonstrated colonization in 40–65% of patients treated with haemodialysis, on average, compared to 17–25% of the general population [17]. It is conceivable that patients on haemodialysis have more frequent colonization by *S. aureus* because they are visiting a hospital/dialysis unit regularly [18].

Several steps should be performed before cannulating an AVF, but these steps are not always based on evidence, and recommendations differ among countries. Several guidelines recommend washing the arm with soap and water at the dialysis unit [2, 16, 19]. However, European guidelines regarding infection control in dialysis oppose this routine, stating that bacterial growth in the normal flora increases when the skin is washed and dried [20]. How to apply the disinfectant has also not been determined definitively. Both back and forth and circular motions are used [16] and discussed in former studies [21–23]. The type of disinfectant also differs among countries. In the US and many parts of Europe, the most common disinfectant is ethanol [8, 24], whereas some countries, such as the UK, Ireland, and Italy, prefer chlorhexidine [24]. Other studies have described povidone-iodine as the most preferred disinfectant [9, 25]. In Sweden, 5 mg/mL chlorhexidine in 70% ethanol is the recommended disinfectant [26] and the most commonly used. Only when contraindicated or when a shortage of chlorhexidine occurs (e.g., during the Covid-19 pandemic) is ethanol used as a substitute [16].

Both local and general AVF infections may lead to major consequences for the patient, such as infectious embolization, endocarditis, and increased risk of mortality [5]. Improved knowledge of factors influencing bacterial growth in the cannulation site is important to decrease the risk of AVF-related infections. Reducing the amount of infections may also decrease hospitalization, antibiotic treatment, and vascular surgical intervention, increasing both patient safety and patient satisfaction [27–29].

Both earlier studies [7, 14] and clinical assumptions from different dialysis units in Sweden have shown that disinfection using chlorhexidine may decrease AVF infections. Thus, this study has the primary aim to compare the outcome of bacterial growth after disinfection using chlorhexidine or ethanol on patients undergoing haemodialysis. The secondary aims were to examine and compare the effects of ethanol and chlorhexidine in combination with arm washing with soap and water immediately before disinfection and explore whether the BH scabs contain the same type of bacteria as found in the patients’ normal skin flora.

## Methods

### Trial design

The trial was a randomized, cross-over, single-blinded clinical trial of four sampling occasions during a 3-month period with an allocation ratio of 1:1. Each sampling occasion was separated by a 2 to 3-week wash-out period to prevent any carry-over effect. The reporting in this paper adheres on CONSORT 2010 statement: extension to randomised crossover trial (Additional file 1) [30].

### Participants, setting, and location

All patients treated with haemodialysis at a dialysis clinic in southeast Sweden who fulfilled the inclusion criteria and did not meet any of the exclusion criteria were asked to participate in this study. Inclusion criteria were age ≥ 18 years, being treated with haemodialysis using an AVF, and BH cannulation using blunt needles. Exclusion criteria were having any type of dialysis catheter (peritoneal dialysis catheter or CVC), previous allergic reaction to the disinfectants used, having been treated with antibiotics during the last 4 weeks, need for an interpreter to understand the study information (written in Swedish), and any kind of cognitive impairment.

**Fig. 1:**
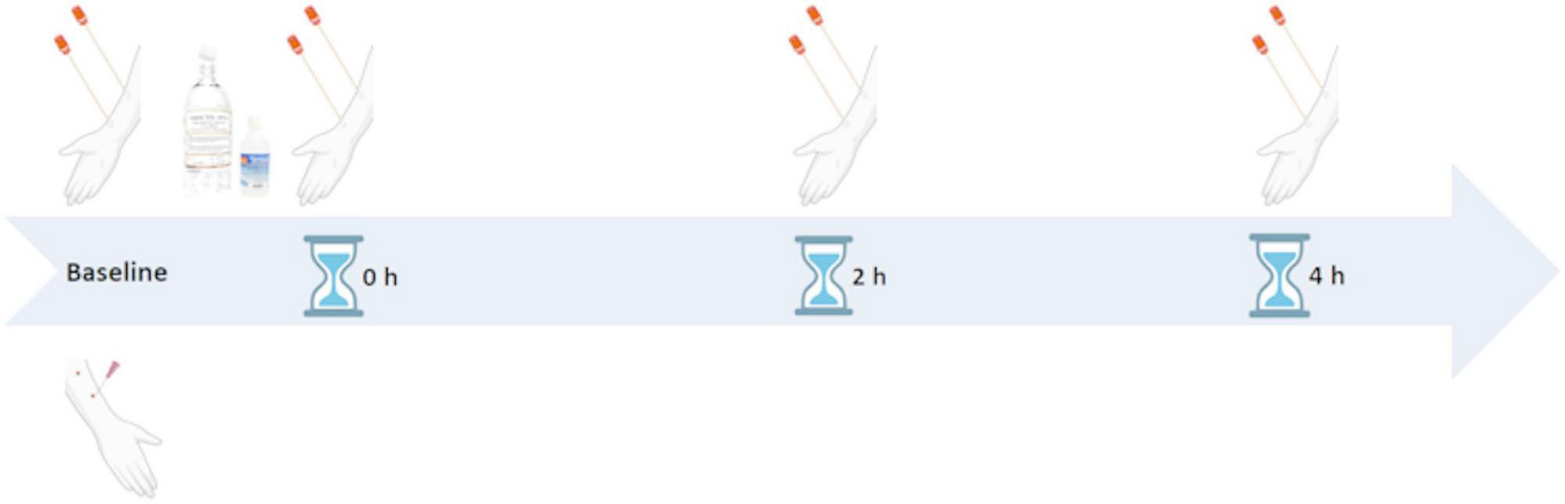
Timeline of the skin preparation and collection of samples. Eight different skin samples were collected during one study visit. Scabs from the buttonhole site were removed and collected. Figure created in www.BioRender.com

### Interventions

**Fig. 2:**
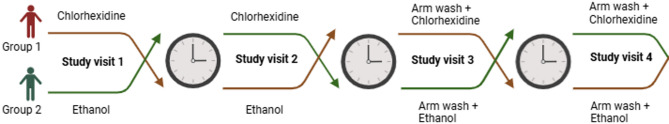
Overview of data collection. Figure created in www.BioRender.com

**Fig. 3:**
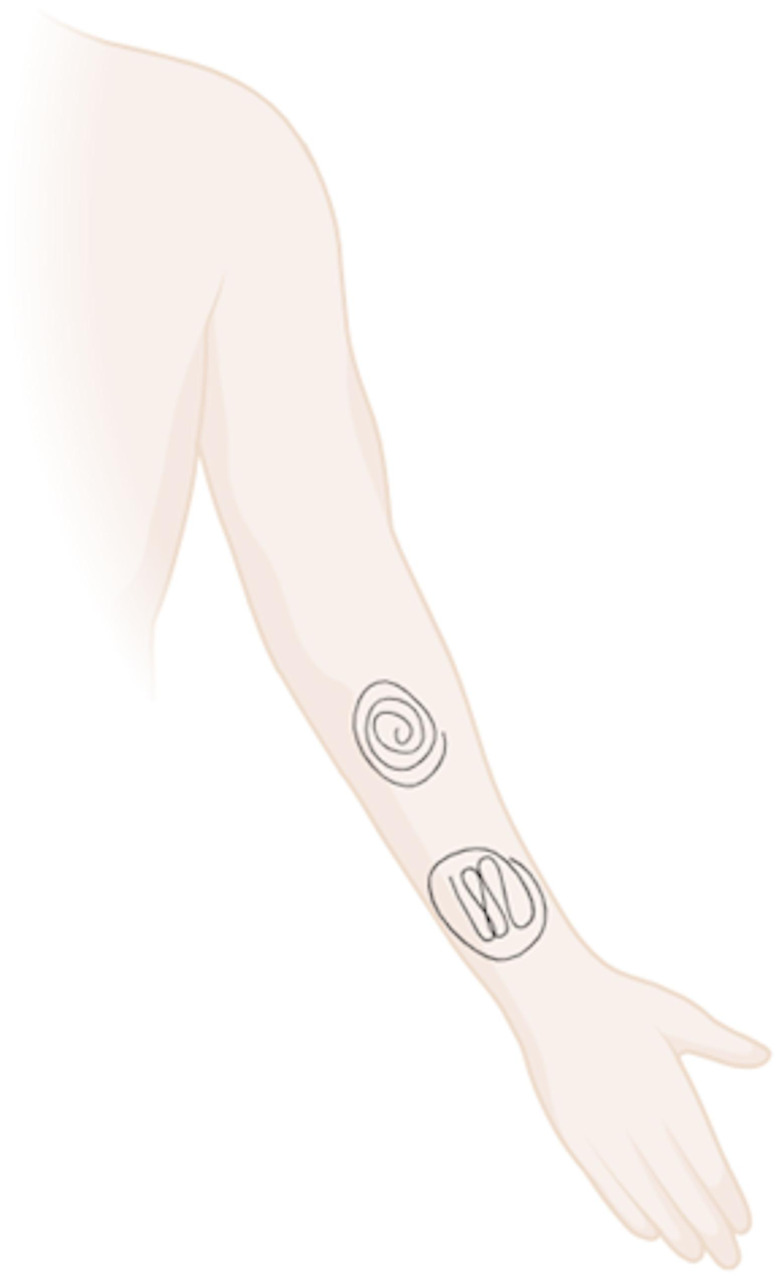
Example of the routine for disinfection of each patient’s arm during the intervention. Figure created in www.BioRender.com

**Fig. 4:**
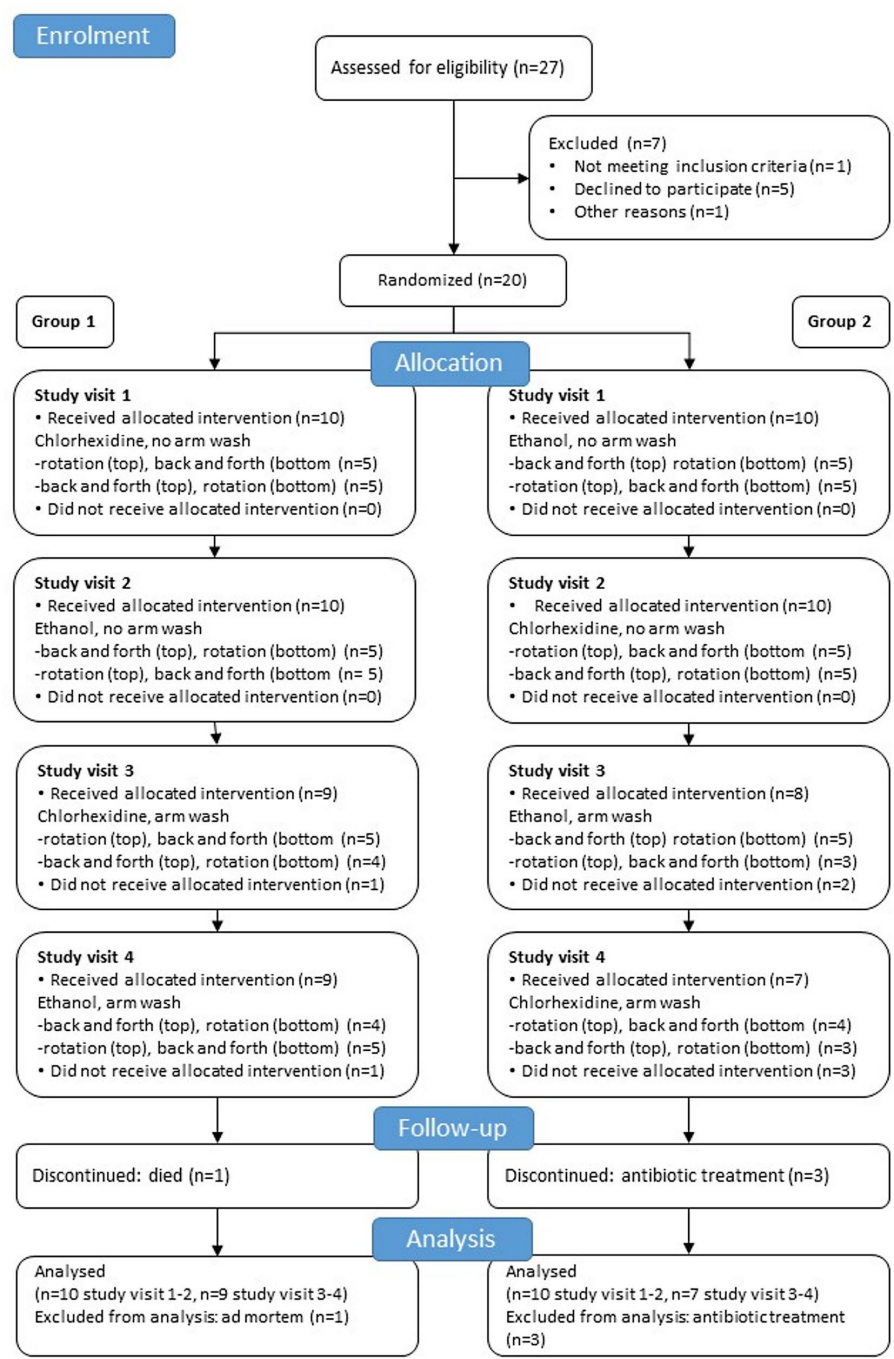
Flowchart of the interventions and participant inclusion

Disinfection was performed on the arm not used for dialysis as the sample method could not be performed without destroying the BH tracks and that the cannulation sites of the patient would not be exposed to disinfectant that might be less effective. All four study visits during the trial were performed during the course of ordinary dialysis treatment. The first sample was obtained just ahead of disinfection. Thereafter, samples were taken directly after the disinfectant effect time, 2 h after disinfection, and 4 h after disinfection (Fig. 1). During the first two study visits, patients were told not to wash the arm, whereas during the last two study visits, they were encouraged to wash the forearm with liquid soap and tap water and thereafter dry it with paper towels for single use. This was done after arriving at the dialysis unit (Fig. 2). Samples were taken using the pencil eraser swab (PES) technique [31] over each disinfected area. Disinfection was performed using either a rotating technique, from the centre and out, or by cleaning back and forth on the upper or lower part of the forearm according to the randomization protocol (Fig. 3). During disinfection, three cotton sponges soaked with disinfectant were used for the upper part of the forearm and three on the lower part of the forearm, each for 10 s. Due to different contact times, recommended by the manufacturers to reach sufficient effect, the skin dried for 30 s after disinfection with chlorhexidine or 2 min after disinfection with ethanol before samples were taken [26, 32]. When the samples were taken, the skin was completely dry. All according to material safety data sheet and the summary of product characteristics [33]. All participants received both types of disinfectant (Fig. 4).

Preparation and cannulations were performed following the ordinary routines of the clinic. After the nurse disinfected the site using 5 mg/mL chlorhexidine in 70% ethanol, scabs were removed from the BH tracks using a blunt 18 G needle, followed by a second disinfection. The nurse that was responsible for the patient´s treatment always wore personal protective equipment (mask, apron, clean gloves) and used a sterile drape under the AVF arm. During the four study visits, the scabs were collected and sampled in the same way as the other samples using eSwab Liquid Amies Preservation Medium (COPAN, Brescia, Italy). Each study visit resulted in nine samples (Fig. 1).

### Outcomes

The samples were kept directly in a fridge (cool bag) until being transported to the microbiological laboratory. At the laboratory, each sample were vortexed for 10 s, spread on blood agar and haematin plates at concentrations of 1:1, 1:10, and 1:100 [34], and kept at 35 °C for 48 h before the number of colony forming units (CFU) was counted. All plates containing 10–200 CFU were evaluated. If more than one plate from each sample contained this amount, the mean number of CFU was used (primary outcome). The exception was the chi-squared analysis, for which ≥ 5 CFU was included, as no plates in the chlorhexidine group had ≥ 10 CFU. Bacteria were identified using matrix-assisted laser desorption/ionization time-of-flight mass spectrometry (MALDI-TOF). If the CFU/mL did not follow a visible natural curve, contamination was suspected, and these samples were excluded from the analysis.

The first author, the last author, and a research nurse from the dialysis unit performed sampling. In the microbiology lab, all samples were assessed by the first author.

### Sample size

A total of 20 patients were included in the study. Earlier research has shown a reduction of CFU to 81.5 ± 3.4% when using 5 mg/mL chlorhexidine in 70% ethanol and to 73.6 ± 5.7% when using 70% ethanol [35]. With a power of 80% and a significance level of 0.05, there should be at least seven patients in each group. To compensate for any losses to follow-up, 10 patients were included in each group.

### Randomization

The first author performed randomization by taking a sealed envelope before the first intervention in each patient. The envelope contained the study protocol, identification number, sequence of disinfection, and whether circular or back and forth arm washing should be performed.

### Blinding

Patients were blinded to the type of disinfectant. Participating nurses and physicians were not blinded for practical reasons.

### Statistical analysis

Per protocol analyses were performed. During each study visit, two samples were taken during each part of the sample series, one from the upper forearm and one from the lower forearm. According to the study protocol, the upper and lower forearm were combined with disinfection in circular or back and forth motions. The outcome from these two measurements were visually compared using median and interquartile range (IQR). As these comparisons were estimated to be similar (Additional file 2), the two measurements were merged and used as a mean. The mean, standard deviation (SD), and percentage of participants were used when study groups were described. The median, IQR, and range were used during group comparisons.

Outcomes from the different interventions were compared using the Wilcoxon signed rank test. In the case of multiple measurements, the significance level was adjusted using the Benjamini-Hochberg method (false discovery rate = 0.1) (primary outcome). The paired t-test was used when comparing baseline measurements before and after arm washing. Chi-squared was used during the bivariate comparison. Cohen´s kappa was used to find similarities between the skin flora and type of bacteria in the scabs (secondary outcome). *P* < 0.05 was considered significant.

Excel 2016 (Microsoft, USA) was used as a database for collecting and grouping data and calculating Cohen´s kappa. Statistical analyses were performed using SPSS Statistics for Windows, version 29 (IBM Corp., Armonk, NY).

### Ethical considerations

All participants provided written consent to participate. The study was performed according to Clinical Trails Regulation (CTR), the Declaration of Helsinki, and ICH-GCP. The study was registered in the Clinical Trials Information System (CTIS) under 2023-505935-11-00 [36]. Ethical approval was given by the Swedish Ethical Review Authority and Swedish Medical Products Agency (Dnr 5.1.1-2023-50761).

**Table 1:** Characteristics of patients

Characteristics of the two randomized groups	Group 1 (*n* = 10)	Group 2 (*n* = 10)
Male, n (%)	8 (80)	8 (80)
Mean age, years ± SD	74.1 ± 10.94	66.4 ± 11.22
Kidney disease, n (%)		
Diabetic nephropathy	3 (30)	2 (20)
Polycystic kidney disease	1 (10)	0 (0)
Hypertension	2 (20)	0 (0)
Glomerulonephritis	1 (10)	2 (20)
Other renal disease	2 (20)	2 (20)
Unknown diagnosis	1 (10)	4 (40)
Immunosuppression*	1 (10)	3 (30)
Cat or dog at home, n (%)	2 (20)	2 (20)
Arm wash frequency, n (%)		
Once a week	3 (30)	2 (20)
2–3 times a week	5 (50)	4 (40)
Once a day	1 (10)	3 (30)
Several times per day	1 (10)	1 (10)

*Cortisone or due to kidney transplantation

## Results

Twenty-seven patients treated with haemodialysis at two dialysis units were asked to participate in this trial (Table 1). Seven of these patients were excluded due to the reasons cited in Fig. 4. Thus, a total of 20 patients were included and randomized during December 2023 and January 2024. Study interventions began directly after randomization and continued until June 2024 according to the study protocol. In the group that started with chlorhexidine, one patient died between study visits two and three due to illness not related to kidney disease or dialysis treatment. In the group that started with ethanol, two patients were excluded after two study visits and one after three study visits due to initiation of antibiotic treatment not related to AVF infections. No serious adverse events or adverse events related to the study procedure occurred during the study.

**Fig. 5:**
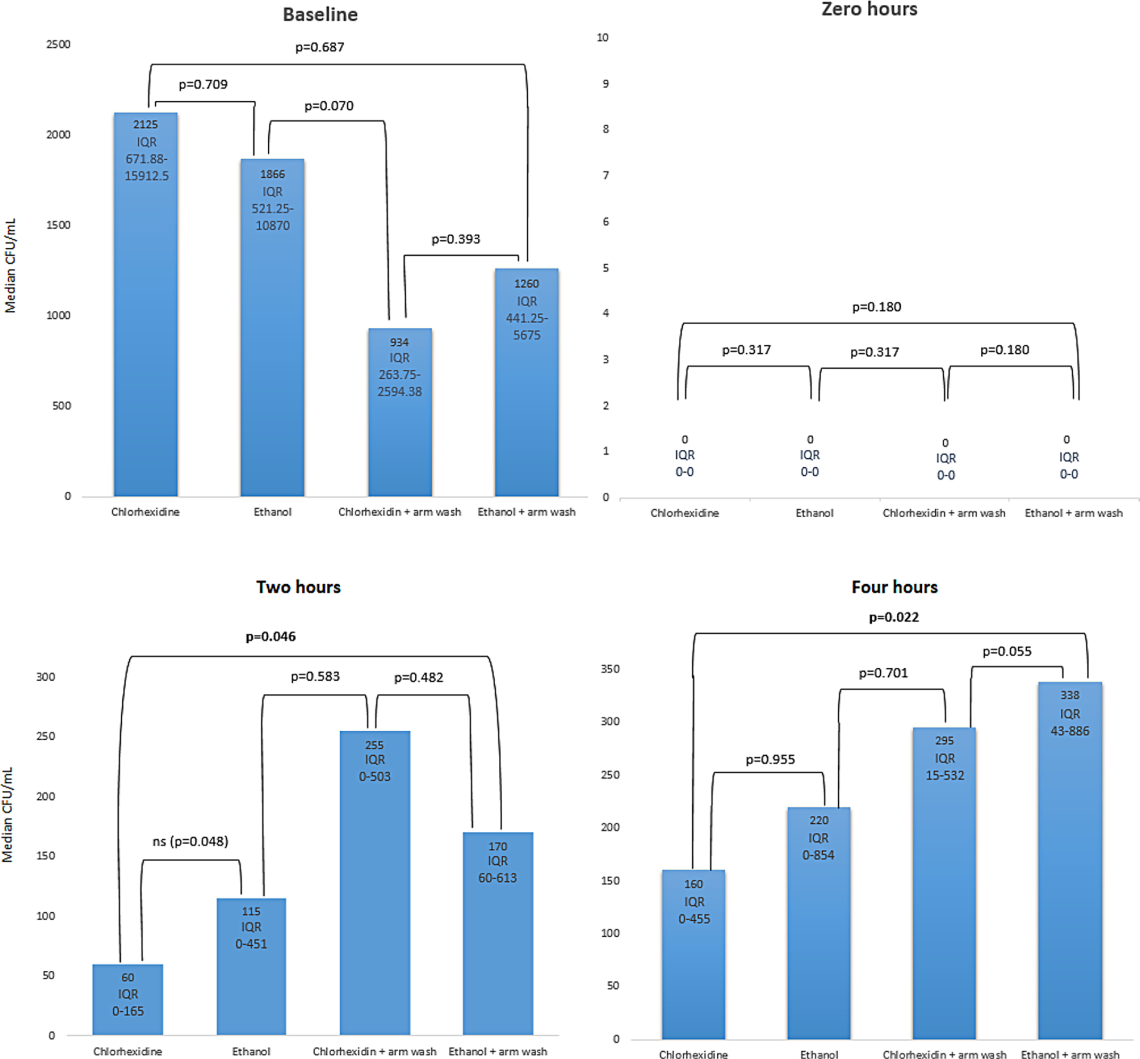
Median CFU/mL at baseline, directly after disinfection, 2 h after disinfection, and 4 h after disinfection. Arm wash, during study visit three and four, was made ahead of baseline sampling. IQR = interquartile range. The significance level was adjusted using the Benjamini-Hochberg method and the critical value is 10%

**Fig. 6:**
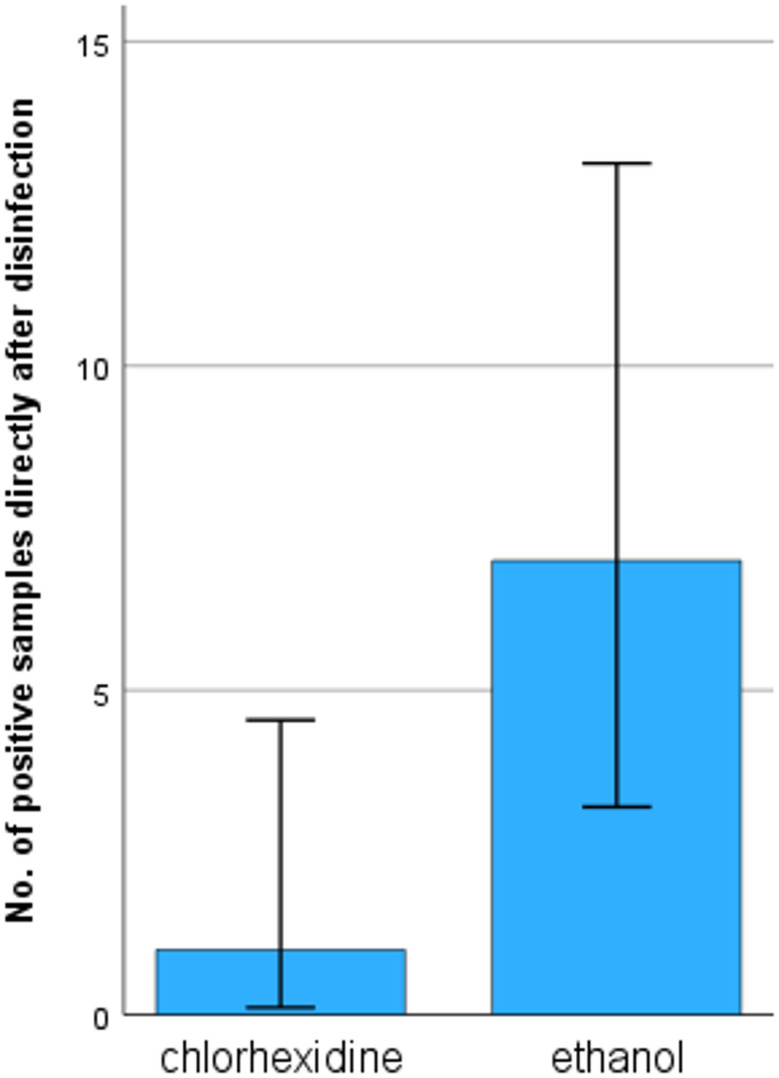
Comparison of positive samples directly after disinfection using chlorhexidine and ethanol. *p* = 0.032. The error bars indicate the 95% confidence interval

The samples taken directly after disinfection showed no CFU on all but three occasions (≥ 10 CFU/mL). These occasions all found after ethanol disinfection; however, they did not affect neither median nor the IQR (Fig. 5). During chi-square of positive (≥ 5 CFU/mL) or negative samples directly after disinfection there was a significant difference (*p* = 0.032), chlorhexidine was positive in one out of 69 occasions (1.4%), ethanol was positive in seven out of 65 occasions (10.8%) (Fig. 6).

When ethanol with arm wash was compared to Chlorhexidine without arm wash, Chlorhexidine had significantly less bacteria after two (*p* = 0.046) and four hours (*p* = 0.022). There were no significant difference if Chlorhexidine with arm wash was compared to ethanol or ethanol in combination with arm wash. (Fig. 5). Neither was it a significant differences in CFU/mL were found between chlorhexidine and ethanol when used without arm washing. There was also not a significant difference when comparing chlorhexidine and chlorhexidine in combination with arm washing or ethanol and ethanol in combination with arm washing (Fig. 5, Additional file 3).

Arm washing significantly decreased the number of bacteria in the whole group when comparing sessions with and sessions without arm washing prior to disinfection. The CFU/mL decreased from 23,997.23 ± 56,459.15 to 3648.74 ± 1218.88 (*p* = 0.047).

Patients’ scabs contained bacteria in a mean 2.3 (± 1.2) cases out of the 4 possible. The types of bacteria in the scabs almost always were found also in the patient’s skin flora (Cohen´s kappa = 0.97). The species most commonly found in the scab was also the most common species in the skin flora from the opposite arm: *Staphylococcus hominis*,* Staphylococcus epidermis*,* Staphylococcus capitis*, *Micrococcus luteus*, and *S. aureus*. Ten of the patients (50%) had *S. aureus* during one to four of the study visits.

## Discussion

The results from this study showed that relatively more bacteria are left on the skin directly after disinfection using ethanol. We also found faster regrowth of the normal skin flora when ethanol was used in combination with arm washing compared to chlorhexidine without arm washing.

Directly after disinfection, more positive samples were seen when ethanol was used. That chlorhexidine is more effective than both ethanol and povidone-iodine has been known for some time [35, 37, 38]. This may be the reason for the increase in infections seen in those who disinfect their BH sites using ethanol [7] or the decrease in infections when chlorhexidine is introduced [14]. Ethanol may be good enough when RL/AP is used, but chlorhexidine may be necessary when BH is used. According to Toma, BH tracks are not the primary reason for the increase in AVF infections [39]. Incomplete disinfection due to a less effective bactericidal agent could be a possible cause of the observed increase in infection rate. If so, recurrent training in hygiene routines [9, 10] and attempts to decolonize cannulation sites using mupirocin [40] may compensate for the shortcomings of disinfection.

There were no significant differences between chlorhexidine and ethanol when compared by CFU/mL as the outcome measurement after application of the disinfectant. This contradicts earlier studies that implied that chlorhexidine is superior to ethanol [35, 41]. However, when arm washing preceded ethanol disinfection, there was a significant difference from chlorhexidine without arm washing after 2 and 4 h. The majority of guidelines regarding cannulation techniques advocate arm washing with soap and water at the dialysis unit before disinfection and cannulation [2, 19, 32]. The recommendations from the European guidelines regarding infection control in dialysis [20] that argue against arm washing ahead of cannulation are more in line with our results. They meant that the moist on the skin might decrease the effect of the disinfectant. Earlier studies also illuminated that handwashing may increase the rate of bacteria [42, 43]. Kaplowitz et al. found that poor hygiene increased the risk of AVF infections [44]. Not performing arm washing in connection with disinfection and cannulation does not have to mean poor hygiene. Our study shows that, regardless of disinfectant and arm washing, the bacteria level decreased to 0 (median) after disinfection. With this in mind, a decrease in bacteria by arm washing immediately adjacent to disinfection may be unnecessary, as the effect of the disinfectant subsides faster than disinfection without arm washing, especially when ethanol and arm washing is compared to chlorhexidine. However, more research is warranted in order to confirm this.

More than half of Swedish dialysis units use circular motions during disinfection ahead of cannulation, whereas back and forth motions are used by almost 25% of Swedish units [16]. European guidelines also describe circular motions as the most commonly used application method, but this is based more on tradition [1]. This study indicates that both application techniques have a similar effect, which is in agreement with earlier studies [1, 45], even though the group size was too small to obtain a valid result in this part of the study.

The importance of disinfection both before and after scab removal is well known [2, 16, 19]. This study adds that bacteria remain in the scab roughly half the time after the first disinfection (with chlorhexidine). This illustrates the importance of disinfection after scab removal. Earlier researchers, who only disinfected before scab removal, noticed a higher incidence of AVF infections [46]. Even though bacteria are found in the scabs, they are a part of the normal skin flora, and only in combination with symptoms may an infection be diagnosed.

The results from this study may have clinical implications. For example, whether it is ethical to use ethanol with the BH technique when we know that it may increase the risk of AVF infection needs to be discussed. In situations in which chlorhexidine is lacking, RL may be used instead. It is also important to illuminate the importance of effect time. During the sampling for this study, the researchers observed that the cannulating nurses differed in both how the disinfectant was applied and the time they let the solution dry afterwards. Therefore, the importance of effect time is valuable to include in education for cannulating dialysis providers. Regular education on needle related hygiene should be organised for both dialysis providers and patients who self-cannulate. Even patients who do not self-cannulate should be informed about all hygiene steps; how to perform them and what the consequences of not following them may be. Knowledge of this will enable patients to remind the dialysis provider of the correct procedure. It is also important to monitor access-related infections and regularly evaluate the results and possible improvements.

This study has some limitations. First, to conduct research with the aim of preventing infections, it would be preferable to use the number of infections as the outcome. However, as the number of participants would be too extensive, this study had to rely on CFU/mL as surrogate rather than a clinical endpoint. It is also not ethical to expose patients to an increased risk of infection as we suspect ethanol would have done. Second, despite the inclusion of the desired numbers of patients, the groups were too small to reach pronounced results. The study also had a single-centre design, which may have affected the outcome. However, in combination with other, similar studies, it may give another piece of the puzzle of how to prevent AVF-related infections. Third, the study was only single-blinded. Fourth, during the study visits, the patient’s free arm was used. During the dialysis treatment, they used this arm without restrictions. For example, some had it as a pillow under the cheek or neck, whereas others covered it with a sleeve. This may have affected the results. However, the majority of samples followed a naturally increasing curve, and those samples that did not (according to contamination) were excluded.

## Conclusions

One of the most important assignments as a dialysis provider is to protect patients from getting infected by their own normal skin flora or surrounding bacteria and other microorganisms. By using 5 mg/mL chlorhexidine in 70% ethanol when the BH cannulation technique is used, the possibility of decreasing the bacteria on the patient’s skin and extending the time to regrowth of normal flora, is greater compared to the use of ethanol. This in turn could possibly lead to fewer AVF infections.

## Electronic supplementary material

Below is the link to the electronic supplementary material.

Supplementary Material 1

Supplementary Material 2

Supplementary Material 3

## Data Availability

The datasets used and analysed during the current study are available from the corresponding author on reasonable request.

## Abbreviations

AP: Area puncture
AVF: Arteriovenous fistula
AVG: Arteriovenous graft
BH: Buttonhole
CFU: Colony Forming Units
CTIS: Clinical Trials Information System
CTR: Clinical Trails Regul
CVC: Central Venous Catheter
IQR: Interquartile range
MALDI-TOF: Matrix-assisted laser desorption/ionization time-of-flight mass spectrometry
PES: Pencil Eraser Swab
RL: Ropeladder
SD: Standard Deviation
