# Supplementary material for: Disinfection with chlorhexidine is more effective than ethanol for buttonhole cannulation in arteriovenous fistula: a randomized cross-over trial
Source: BMC Nephrol. 2025 Jul 19;26:402. doi: 10.1186/s12882-025-04230-z (PMC12275420; doi:10.1186/s12882-025-04230-z)
Supplement: Supplementary file 2 — Supplementary Material 2 [file 12882_2025_4230_MOESM2_ESM.docx]

| *Additional file 2*: Comparison of median CFU on the upper or lower forearm and during disinfection using circular or back and forth motions. | | | | | |
| --- | --- | --- | --- | --- | --- |
|  | | **Chlorhexidine** | **Ethanol** | **Chlorhexidine + arm wash** | **Ethanol + arm wash** |
| **Baseline** | Upper forearm | 2600 (435 – 6475) | 1700 (563 – 9300) | 815 (250 – 3425) | 1795 (633 – 5650) |
|  | Lower forearm | 1710 (638 – 9563) | 1720 (480 – 3750) | 920 (198 – 1351) | 995 (260 – 1515) |
| **Directly after** | Upper forearm | 0 (0 – 0) | 0 (0 – 0) | 0 (0 – 0) | 0 (0 – 0) |
|  | Lower forearm | 0 (0 – 0) | 0 (0 – 0) | 0 (0 – 0) | 0 (0 – 0) |
| **2 hours after** | Upper forearm | 0 (0 – 205) | 55 (0 – 310) | 85 (0 – 545) | 140 (0 – 885) |
|  | Lower forearm | 0 (0 – 150) | 155 (0 – 568) | 185 (0 – 721) | 220 (120 – 530) |
| **4 hours after** | Upper forearm | 130 (0 - 403) | 0 (0 – 881) | 295 (0 – 518) | 460 (0 – 1413) |
|  | Lower forearm | 100 (0 – 355) | 110 (0 – 530) | 330 (0 – 641) | 295 (0 – 650) |
| **Baseline** | Rotating | 2820 (605 – 9563) | 1938 (420 – 11175) | 865 (188 – 2844) | 1020 (543 – 3750) |
|  | Back and forth | 1910 (448 – 6475) | 1730 (515 – 3750) | 915 (280 – 1704) | 1025 (380 – 3850) |
| **Directly after** | Rotating | 0 (0 – 0) | 0 (0 – 0) | 0 (0 – 0) | 0 (0 – 0) |
|  | Back and forth | 0 (0 – 0) | 0 (0 – 0) | 0 (0 – 0) | 0 (0 – 0) |
| **2 hours after** | Rotating | 0 (0 – 150) | 145 (0 – 905) | 120 (0 – 560) | 220 (0 – 585) |
|  | Back and forth | 120 (0 – 288) | 55 (0 – 293) | 140 (0 – 800) | 150 (28 – 475) |
| **4 hours after** | Rotating | 110 (0 – 323) | 100 (0 – 930) | 375 (125 – 613) | 350 (55 – 1323) |
|  | Back and forth | 110 (0 – 410) | 110 (0 – 440) | 270 (0 – 660) | 260 (0 – 873) |

## The median value is given with the interquartile range in parentheses.
