# Supplementary material for: Disinfection with chlorhexidine is more effective than ethanol for buttonhole cannulation in arteriovenous fistula: a randomized cross-over trial
Source: BMC Nephrol. 2025 Jul 19;26:402. doi: 10.1186/s12882-025-04230-z (PMC12275420; doi:10.1186/s12882-025-04230-z)
Supplement: Supplementary file 3 — Supplementary Material 3 [file 12882_2025_4230_MOESM3_ESM.docx]

| *Additional file 3:* Measurement of CFU/mL on the arm at baseline, directly after disinfection, 2 hours after disinfection, and 4 hours after disinfection. | | | |
| --- | --- | --- | --- |
| **Comparison between chlorhexidine vs ethanol** | | | |
|  | **CFU/mL chlorhexidine** | **CFU/mL ethanol** | **p-value** |
| **Baseline** | 2125 (100)  IQR  671.88-15,912.5  Range  220-119,000 | 1866.25 (100)  IQR  521.25-10,870  Range  1-251,500 | 0.709 |
| **Directly after** | 0 (0)  IQR  0-0  Range  0-0 | 0 (0)  IQR  0-0  Range  0-220 | 0.317 |
| **2 hours** | 60 (2.82)  IQR  0-165  Range  0-533 | 115 (6.16)  IQR  0-451.25  Range  0-1160 | 0.048 |
| **4 hours** | 160 (7.53)  IQR  0-455  Range  0-1193 | 220 (11.79)  IQR  0-2085  Range  0-853.5 | 0.955 |
| **Comparison between chlorhexidine and arm wash vs ethanol and arm wash** | | | |
|  | **CFU/mL chlorhexidine and arm wash** | **CFU/mL ethanol and arm wash** | **p-value** |
| **Baseline** | 933.75 (100)  IQR  263.75-2594.38  Range  120-7500 | 1260 (100)  IQR  441.25-5675  Range  1-28,610 | 0.393 |
| **Directly after** | 0 (0)  IQR  0-0  Range  0-0 | 0 (0)  IQR  0-0  Range  0-115 | 0.180 |
| **2 hours** | 255 (27.31)  IQR  0-503.13  Range  0-1422.5 | 170 (13.49)  IQR  60-612.5  Range  0-3600 | 0.480 |
| **4 hours** | 295 (31.59)  IQR  15-531.79  Range  0-1652.5 | 337.5 (26.79)  IQR  42.5-886.25  Range  0-5703 | 0.055 |
| **Comparison between chlorhexidine vs chlorhexidine and arm wash** | | | |
|  | **CFU/mL chlorhexidine** | **CFU/mL chlorhexidine and arm wash** | **p-value** |
| **Baseline** | 2125 (100)  IQR  671.88-15,912.5  Range  220-119,000 | 933.75 (100)  IQR  263.75-2594.38  Range  120-7500 | 0.224 |
| **Directly after** | 0 (0)  IQR  0-0  Range  0-0 | 0 (0)  IQR  0-0  Range  0-0 | 1.00 |
| **2 hours** | 60 (2.8)  IQR  0-165  Range  0-533 | 255 (27.31)  IQR  0-503.13  Range  0-1422.5 | 0.87 |
| **4 hours** | 160 (7.5)  IQR  0-455  Range  0-1193 | 295 (31.59)  IQR  15-531.79  Range  0-1652.5 | 0.6 |
| **Comparison between ethanol vs ethanol and arm wash** | | | |
|  | **CFU/mL ethanol** | **CFU/mL ethanol and arm wash** | **p-value** |
| **Baseline** | 1866.25 (100)  IQR  521.25-10,870  Range  1-251,500 | 1260 (100)  IQR  441.25-5675  Range  1-28,610 | 0.177 |
| **Directly after** | 0 (0)  IQR  0-0  Range  0-220 | 0 (0)  IQR  0-0  Range  0-115 | 1.00 |
| **2 hours** | 115 (6.2)  IQR  0-451.25  Range  0-1160 | 170 (13.5)  IQR  60-612.5  Range  0-3600 | 0.126 |
| **4 hours** | 220 (11.8)  IQR  0-2085  Range  0-853.5 | 337.5 (26.8)  IQR  42.5-886.25  Range  0-5703 | 0.638 |
| **Comparison between ethanol vs chlorhexidine and arm wash** | | | |
|  | **CFU/mL ethanol** | **CFU/mL chlorhexidine and arm wash** | **p-value** |
| **Baseline** | 1866.25 (100)  IQR  521.25-10,870  Range  1-251,500 | 933.75 (100)  IQR  263.75-2594.38  Range  120-7500 | 0.070 |
| **Directly after** | 0 (0)  IQR  0-0  Range  0-220 | 0 (0)  IQR  0-0  Range  0-0 | 0.317 |
| **2 hours** | 115 (6.16)  IQR  0-451.25  Range  0-1160 | 255 (27.3)  IQR  0-503.13  Range  0-1422.5 | 0.583 |
| **4 hours** | 220 (11.79)  IQR  0-2085  Range  0-853.5 | 295 (31.6)  IQR  15-531.79  Range  0-1652.5 | 0.701 |
| **Comparison between chlorhexidine vs ethanol and arm wash** | | | |
|  | **CFU/mL chlorhexidine** | **CFU/mL ethanol and arm wash** | **p-value** |
| **Baseline** | 2125 (100)  IQR  671.88-15,912.5  Range  220-119,000 | 1260 (100)  IQR  441.25-5675  Range  1-28,610 | 0.687 |
| **Directly after** | 0 (0)  IQR  0-0  Range  0-0 | 0 (0)  IQR  0-0  Range  0-115 | 0.180 |
| **2 hours** | 60 (2.8)  IQR  0-165  Range  0-533 | 170 (13.5)  IQR  60-612.5  Range  0-3600 | ***0.046*** |
| **4 hours** | 160 (7.5)  IQR  0-455  Range  0-1193 | 337.5 (26.8)  IQR  42.5-886.25  Range  0-5703 | ***0.022*** |
| The values are given as the median with the percentage of patients in parentheses, followed by the interquartile range (IQR) and range. The significance level was adjusted using the Benjamini-Hochberg method and the critical value is 10%. | | | |
